# Immunizing against Anogenital Cancer: HPV Vaccines

**DOI:** 10.1371/journal.ppat.1005587

**Published:** 2016-05-19

**Authors:** Cloe S. Pogoda, Richard B. S. Roden, Robert L. Garcea

**Affiliations:** 1 Department of Molecular, Cellular, and Developmental Biology, University of Colorado at Boulder, Boulder, Colorado, United States of America; 2 Department of Pathology, Johns Hopkins University, Baltimore, Maryland, United States of America; University of Michigan Medical School, UNITED STATES

## Human Papillomaviruses

Most of us are familiar with foot and hand warts caused by the skin tropic human papillomaviruses (HPV). However, it is less well recognized that infection with HPV types that replicate in the anogenital mucosa is the most prevalent sexually transmitted disease in the world. Indeed, a recent estimate suggests that 80%–90% of the sexually active population is exposed during their lifetime to mucosal HPV [1]. Of the more than 40 genotypes of mucosal HPV, most result in benign and transient disease, such as genital warts caused by HPV types 6 and 11. However, 15 HPV genotypes (e.g., HPV types 16 and 18) are classified as high-risk because they are causal agents of human cancers [2]. Almost all of the half million cervical cancer cases that occur worldwide each year are related to infection with high-risk HPV (hrHPV) [4]. High-risk HPVs, most especially HPV16, are also implicated in the development of some anal, oropharyngeal, vaginal, vulval, and penile cancers. Within a given population, the most prevalent hrHPV genotype varies with geographical location [1–3]. Combined, all of the hrHPVs are responsible for approximately 5% of all cancers worldwide (10% in women) [1–3]. HPV infection is a significant health burden on the United States economy, costing approximately US$7.6 billion per year for screening (Pap smear) and cervical cancer treatment [4]. Recommendations for the current vaccines are for routine inoculation of females 13–26 years of age as well as males 13–21 years of age. The vaccines are most effective when administered prior to potential virus exposure (i.e., before commencement of sexual activity) [5]. Vaccination is the most effective strategy to prevent these cancers and associated morbidities [3–5].

## First Generation Vaccines

The non-enveloped virus capsid is comprised of 72 pentamers (capsomeres) of the major capsid protein L1, arranged into a T = 7*d* icosahedral lattice, with an additional 12–36 L2 proteins located within the capsid shell [6]. The L1 protein, prepared by recombinant yeast (Merck) or baculovirus (GlaxoSmithKline [GSK]) expression, spontaneously self-assembles into virus-like particles (VLPs) [3–7]. L1 VLPs, which lack both the L2 structural protein and the infectious virus genome, are highly immunogenic. Vaccination with L1 VLPs effectively prevents infection [7–11]. However, protection for the most part is type restricted, thus vaccines including several L1 VLPs of the most medically significant HPV genotypes have been developed. Currently there are three prophylactic vaccines available: Gardasil—tetravalent targeting HPV types 6, 11, 16, and 18 (Merck); Gardasil 9—nonavalent targeting HPV 6, 11, 16, 18, 31, 33, 45, 52, and 58 (Merck); and Cervarix—bivalent against HPV 16 and 18 (GlaxoSmithKline) [7,8]. These vaccines are formulated with alum-based adjuvants: Merck’s vaccines contain amorphous aluminum hydroxyphosphate sulfate, while GSK uses AS04 (which contains both 3-O-desacyl-4-monophosphoryl lipid A [MPL] and aluminum hydroxide) [10]. Both Gardasil and Cervarix target the hrHPVs responsible for 70% of cervical cancers, and Gardasil 9 targets up to 90% (both Gardasil vaccines also target two HPV types responsible for ~90% of genital warts) [11]. These vaccines offer only prophylactic protection and have no demonstrated therapeutic effect for treating existing HPV infections.

Gardasil has been in clinical use since 2008, Cervarix since 2009, and Gardasil 9 since 2014. While these vaccines are safe and effective, they have limitations that may restrict their use, especially in developing countries. The foremost is cost, which is approximately US$450 (Gardasil) and US$495 (Gardasil 9) in the US for the complete course of three injections. Another is the requirement for multiple doses and the reality of patient non-compliance for boosts [12]. Finally, refrigeration is required during shipping and storage, posing logistical problems in areas lacking appropriate infrastructure. The Global Alliance for Vaccines and Immunization (GAVI) is working towards providing 30 million women in Southeast Asia and sub-Saharan Africa protection against HPV by 2020. They have secured a reduced pricing agreement of approximately US$5 per dose from Merck and GSK. It is unclear, however, whether it will be feasible to continue to provide these lowered-cost doses without GAVI support. An alternative would be to develop second generation vaccines that can be manufactured locally and for less cost.

## Second Generation Vaccines

To address the high cost, holes in the breadth of protection, multi-dose requirements, and storage temperature limitations inherent in the current vaccines, second generation vaccines are being developed.

A major obstacle to new vaccine formulations is the cost required for their clinical development, testing, and manufacture. To demonstrate effectiveness against hrHPV, clinical trials must show a statistical reduction in histopathologically confirmed Cervical Intraepithelial Neoplasia (CIN)2+. The ability to identify a protective level of antibody in serum or other such biomarkers of protection could potentially help reduce costs. If a minimal protective titer could be defined, the pseudovirus neutralization assay (pseudovirus is the papillomavirus, in which the infectious genome has been replaced with an internal reporter plasmid) could be used to rapidly optimize potential vaccine candidates by allowing assessment and validation of their ability to induce and maintain protective responses [13]. The assay, which measures how effectively antibodies block infection by pseudoviruses, can determine not only the presence but also the ability of antibodies to neutralize the antigen. There is also interest in manufacturing using low-cost expression systems, such as bacteria, for the production of L1-based vaccines. As multivalent formulations are still required for broad protection, production of a single broadly protective antigen would potentially simplify production and further reduce manufacturing costs.

## L2 Antigens

One promising alternative to VLP antigens is a subdominant neutralizing epitope in the L2 protein of the virus [14]. A linear neutralizing epitope at the amino terminus of L2 is exposed while the virus resides on the basement membrane during infection, and this epitope is generally well conserved across HPV genotypes [14]. As there is some sequence diversity within this L2 region for different HPV genotypes, one strategy has been to fuse the N-terminal regions of L2s from several HPV genotypes and express a concatemeric peptide (e.g., in *Escherichia coli*) [14]. This L2 antigen has been shown to protect against infectious challenge in animal models [14]. Unfortunately, L2 fusions, even when injected with adjuvants, have low immunogenicity in comparison with VLPs when measured by neutralizing antibody titers [15]. An alternative strategy to increase the L2 immune response has been to incorporate the epitopes into exposed loops of L1-VLPs. This approach combines the advantages of both L1- and L2-specific protective responses [14]. While these vaccines have the potential to be less expensive, a major hurdle remains: demonstrating increased or comparable safety and efficacy against HPV infection when compared to the already available vaccines [15].

## Capsomere Antigens

Another alternative antigen is the L1 pentameric subunit or capsomere that retains necessary neutralizing epitopes to induce an immune response against HPV [16]. Capsomeres can be purified after expression in *E*. *coli*, which may represent a significant manufacturing cost reduction. Animal studies have demonstrated that HPV capsomeres alone induce lower antibody titers when compared to VLPs. However, when injected with an adjuvant they protect against infection and yield equivalent neutralizing antibody titers [16]. Additionally, the pentamers can be effectively lyophilized to increase thermostability, resulting in formulations that can be shipped and stored without refrigeration [17]. Thus, the potential low cost of production and thermostability make capsomeres an attractive possibility for a second generation HPV vaccine [17].

## Therapeutic Vaccines

An improvement to the current prophylactic HPV vaccines would be the addition of therapeutic properties with the potential to treat currently infected patients [18]. Numerous different strategies have been examined in candidate therapeutic HPV vaccines, but only a few have been combined with prophylactic antigens [18]. Since the capsid antigens (L1 and L2) are not expressed in the basal epithelial cells that harbor persistent HPV infections, therapeutic vaccines aim to elicit cytotoxic T cell responses against the HPV early viral gene products E1, E2, E5, E6, and/or E7 [18]. E6 and E7 are most often targeted because they are expressed by all HPV-infected cells and are required for the viability of cancer cells [18].

Initial efforts to generate a combination preventive–therapeutic antigen centered on fusions of L2 with E7 (e.g., HPV6 L2E7, termed “TA-GW,” which was tested for the treatment of genital warts), or both E6 and E7 (e.g., HPV16 L2E7E6, termed “TA-CIN,” which was tested in healthy volunteers, as well as for the treatment of HPV16-associated high-grade anogenital intraepithelial neoplasia). While these vaccines showed promise in some early clinical trials, their therapeutic effectiveness has not been demonstrated [18].

Early viral antigens have also been incorporated into VLPs (and capsomeres) by their fusion to L1. These vaccines were tested in clinical trials and were effective at producing a prophylactic response as well as a cellular response. However, the cellular response failed to correlate with reduction of anogenital intraepithelial neoplasia [18].

DNA vaccines are also being explored, with the positive attributes of being considered safe, easy to produce, and having inherent adjuvant properties [18,19]. These vaccines, which are anticipated to be genotype specific, are in ongoing Phase I clinical trials and still need to demonstrate clinical significance in clearing HPV infections and associated dysplastic lesions. Early clinical studies suggest that the presence of circulating tumor-specific cytotoxic lymphocyte cells may not be sufficient to guarantee therapeutic success in patients [19].

## Future Outlook

Vaccines have remarkable potential to prevent cancers that are related to infectious agents (e.g., HPV and Hepatitis B). While the latest HPV vaccine offers protection against up to 90% of cervical cancer, next generation vaccines will potentially offer broader protection and be more practical for universal implementation. Hopefully, they will address the issues of cost by using alternative production systems, fewer but more cross-protective antigens (L1 or L2), and suitability for manufacture in the regions where the vaccines will be delivered. By utilizing techniques such as lyophilization, these new vaccines may be shipped and stored without refrigeration. New delivery methods, such as nanoparticle platforms, have the potential to eliminate the need for multiple doses through timed-release technology [20]. More stable formulations also create the potential for aerosol or patch deliverable vaccines to eliminate the need for needles. Second generation vaccines may even have therapeutic properties that treat existing HPV infections. These factors may alter the current guidelines regarding when and to which populations vaccines should be administered. As current vaccines are administered, it will be important to monitor if an increase of non-targeted hrHPV genotypes occur. This potential viral replacement may dictate that second generation vaccines must immunize against different strains or be more broadly effective. As the current and second generation vaccines continue to evolve and are used by a greater fraction of the global population, we look forward to seeing the decreasing rates of anogenital (and likely oropharyngeal) cancers and deaths due to HPV infection.
